# Sol-gel Entrapped *Candida antarctica* lipase B — A Biocatalyst with Excellent Stability for Kinetic Resolution of Secondary Alcohols

**DOI:** 10.3390/molecules171113045

**Published:** 2012-11-02

**Authors:** Anca Ursoiu, Cristina Paul, Tibor Kurtán, Francisc Péter

**Affiliations:** 1 Faculty of Industrial Chemistry and Environmental, University “Politehnica” of Timisoara, C. Telbisz 6, 300001 Timisoara, Romania; Email: anca.ursoiu@chim.upt.ro (A.U.); cristina.paul@chim.upt.ro (C.P.); 2 Department of Organic Chemistry, University of Debrecen, Debrecen 4032, Egyetem tér 1, Hungary; Email: kurtan.tibor@science.unideb.hu

**Keywords:** lipase, sol-gel immobilization, ionic liquids, additive, reusability, stability, kinetic resolution

## Abstract

Sol-gel entrapment is an efficient immobilization technique that allows preparation of robust and highly stable biocatalysts. Lipase from *Candida antarctica* B was immobilized by sol-gel entrapment and by sol-gel entrapment combined with adsorption on Celite 545, using a ternary silane precursor system. After optimization of the immobilization protocol, the best enzyme loading was 17.4 mg/g support for sol-gel entrapped lipase and 10.7 mg/g support for samples obtained by entrapment and adsorption. Sol-gel immobilized enzymes showed excellent values of enantiomeric ratio E and activity when ionic liquid 1-octyl-3-methyl-imidazolium tetrafluoroborate was used as additive. Immobilization increased the stability of the obtained biocatalysts in several organic solvents. Excellent operational stability was obtained for the immobilized lipase, maintaining unaltered catalytic activity and enantioselectivity during 15 reuse cycles. The biocatalysts were characterized using scanning electron microscopy (SEM) and fluorescence microscopy. The improved catalytic efficiency of entrapped lipases recommends their application for large-scale kinetic resolution of optically active secondary alcohols.

## 1. Introduction

The use of biocatalytic reactions is an important approach to synthesize pharmaceutical products and fine chemicals [1,2,3,4]. Biotechnology offers many possibilities for new medical therapies and the manufacture of new pharmaceuticals, including the manufacture of small-molecule pharmaceuticals via biocatalysis [5]. 

Reactions catalyzed by various types of hydrolases are predominant among biotransformations. Lack of sensitive cofactors, which have to be recycled, makes them particularly attractive for organic synthesis. Among hydrolytic enzymes lipases and esterases are frequently used because they accept a broad range of substrates and often exhibit high enantioselectivity. There are a large number (>50) of commercial lipases and esterases currently available for biocatalytic reactions. Lipase-catalyzed reactions in organic solvents are becoming increasingly important in enantioselective synthetic chemistry, as certain reactions which are sensitive to water can be carried out in organic media [6,7,8,9,10,11,12]. The kinetic resolution of racemic mixtures represents one of the most significant applications of lipase biocatalysis, since the resulting enantiomers represent important building blocks for the pharmaceutical industry [13].

Synthetic application of isolated enzymes being native proteins suffers several drawbacks. Proteins are relatively unstable in water and the recovery of the enzymes from aqueous reaction mixtures may be difficult due to the high solubility. Moreover, the difficult recovery of the enzymes may render the process uneconomical [14]. The easiest solution to all these drawbacks is enzyme immobilization [1,2,3,14]. Among the multitude of the immobilization methods, including adsorption, covalent attachment and entrapment within polymers, the entrapment of enzymes in organic/inorganic hybrid polymer matrices has received much attention and has provided new opportunities in the field of material science. Mesoporous silica supports were proven particularly efficient for applications related to retention and release of various biomolecules [15]. Sol-gel encapsulation has proved to be a particularly easy and effective way to immobilize purified enzymes and other proteins or even whole cells [1,3,14,16,17,18,19,20,21,22]. The main advantages of this immobilization technique are enhanced operational and thermal stability of the obtained preparates. The sol-gel immobilized lipases can be supported on inert materials such as Celite to improve the diffusion of the substrates or products to and from the enzyme [19,23]. 

Ionic liquids (ILs) are organic salts that melt below 100 °C. The interest in ILs comes from their potential as ‘green solvents’ because of their non-volatile character and thermal stability, which makes them potentially attractive alternatives for volatile organic solvents. Specifically, the interesting property of ILs as lipase stabilizers is their insolubility in hydrophobic organic solvents, because lipases are usually used in organic solvents to carry out various synthetic reactions. Moreover, IL-coated lipases showed significantly enhanced activity in organic solvents [24]. 

The interesting property of IL as an additive in sol-gel immobilization process is their insolubility in hydrophobic organic solvents, and ILs coimmobilized with enzymes in silica can increase the activity and stability of enzymes [25]. 

There are several advantages of carrying out biocatalytic processes in organic media, such as better solubility of substrates and easier recovery of product/enzyme. Moreover, the enantioselectivity in organic solvents is often higher than that of corresponding hydrolytic reaction in water. However, this process could also have some drawbacks, like as a slower reaction rate and possible decrease of optical purity of the desired product due to the reversible nature of the process [6]. For this reason, it is important to recognize the effect of organic solvents on the employed biocatalyst.

The basidiomyceteous yeast *Candida antarctica* produces two different lipases, named A and B. CaLB exhibits a very high degree of substrate selectivity both with respect to regioselectivity and enantioselectivity. For CaLB, however, the most extensive area of use is in the resolution of racemic alcohols, amines and acids, or the preparation of optically active compounds from *meso* reactants [26].

The main goal of this study was to obtain robust solid-phase biocatalysts with high activity, enantioselectivity, and stability for optical resolution of secondary alcohols, using the sol-gel technique. The obtained preparations were evaluated in transesterification reactions with vinyl acetate, this reaction allowing high yields and easier shift of the equilibrium towards synthesis than in case of esterification.

## 2. Results and Discussion

### 2.1. Influence of Enzyme Loading on Activity and Enantioselectivity of Sol-Gel Entrapped Lipase

**Figure 1:**
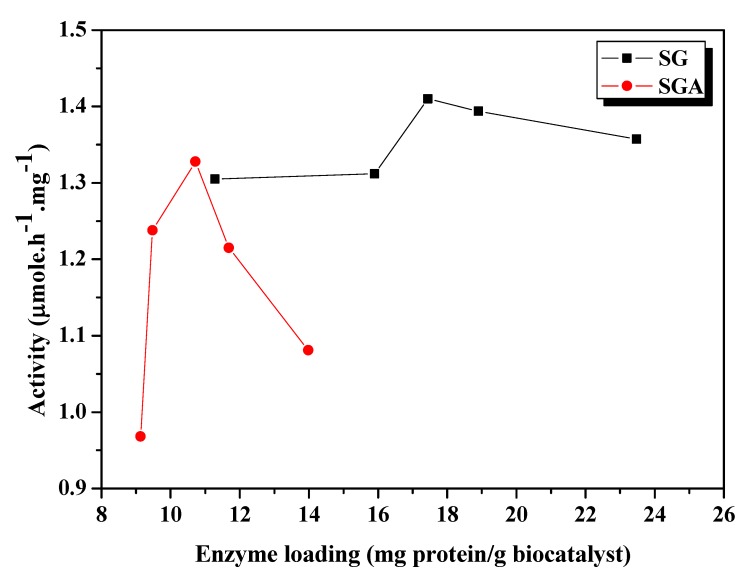
Influence of enzyme loading on catalytic activity of CaLB lipase immobilized by sol-gel entrapment (SG) or sol-gel entrapment combined with adsorption (SGA), in the acylation of 2-octanol at 40 °C, calculated at 6h reaction time.

**Figure 2:**
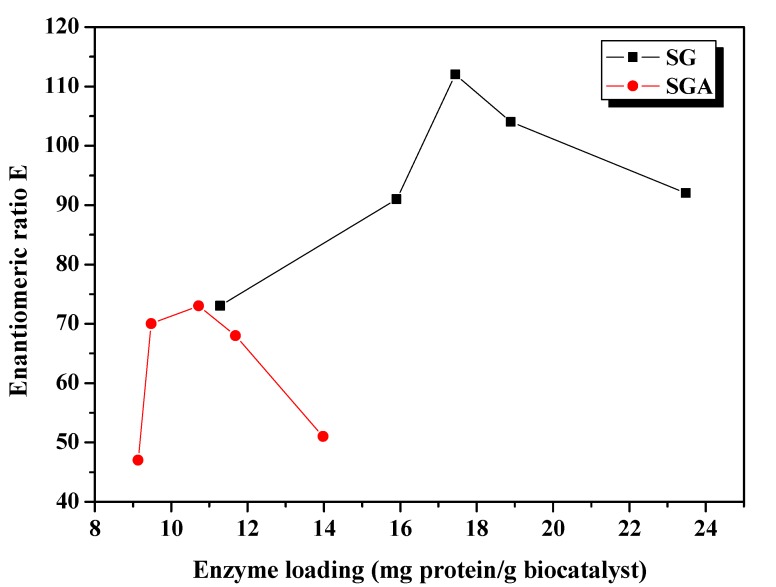
Influence of enzyme loading on the enantioselectivity of CaLB lipase immobilized by sol-gel entrapment (SG) or sol-gel entrapment combined with adsorption (SGA), in acylation of 2-octanol at 40 °C, calculated at 6 h reaction time.

The physical properties of the sol-gel matrix are very important, as they determine the final properties of entrapped enzymes. Pore parameters and particle size determine the total surface area and thus affect the immobilization capacity. Nonporous supports show few diffusional limitations but have lower loading capacity. Consequently, porous supports are generally preferred because of the higher surface area that allows better enzyme loading. Enzyme loading capacity is an essential parameter of immobilization, since the price of the enzyme often represents the main factor hindering process scale-up. Obviously, higher enzyme loadings should result in higher activity, but not all loaded enzyme will remain active, e.g., molecules entrapped inside narrow pores may not be available for the substrate. Another possible drawback could be physical or chemical denaturation of the enzyme during the immobilization process. Thus, high protein loadings must be correlated with high residual activity of the immobilized enzyme. The aim of this study was to find the best enzyme loading capacity in the sol-gel matrix for the investigated immobilization techniques. In this order, different amounts of lipase B from *Candida antarctica* B were immobilized by sol-gel entrapment or by entrapment combined with adsorption on Celite 545 [19]. The sol-gel matrix was obtained using a ternary precursor system with PhTMOS, MeTMOS and TMOS at 1.6:0.4:1 molar ratio, and [Omim]BF_4_ as an immobilization additive [17]. The immobilized lipases have been evaluated in the model reaction of enantioselective acylation of 2-octanol with vinyl acetate at 40 °C, in *n*-hexane. The loaded protein was calculated as the difference between the protein contained in the native enzyme subjected to immobilization and the protein remained in the washing solutions after immobilization, related to the amount of the dried sol-gel preparation. In our case, the immobilization yield of protein, probably representing the limit of this method, was about 80%, regardless to the protein amount subjected to immobilization. Part of the enzyme remained in the solution that was eliminated during the washing step, after gelation. As a result, the loaded protein (represented on the abscissa of Figure 1 and Figure 2) increased with the amount of enzyme subjected to immobilization, but not all encapsulated enzyme remained active at the end of this process.

As shown in Figure 1, the highest activity value among the sol-gel entrapped preparates has been registered at 17.4 mg/g enzyme loading. Overloading above this optimum value resulted in decrease of activity. Other authors also observed that catalytic activity of an immobilized lipase system was not always correlated with the enzyme loading [27,28]. 

For immobilization by the combined method of sol-gel entrapment and adsorption the same enzyme amounts were used, but the measured protein loadings were lower (between 9.1 and 13.4 mg/g), due to the higher weight of the obtained preparation that contained the support material (Celite), as well. The highest value of transesterification activity was achieved at 10.7 mg/g enzyme loading, and the loaded protein profile was much sharper than in case of simple sol-gel entrapment (Figure 1). Probably, an increase of the amount of enzyme subjected to immobilization beyond the optimal value results in retention of part of the protein in the inner pores of the adsorbent material and a diminuation of its catalytic activity by steric hindrance or mass transfer limitations.

The activity values for the best immobilized preparates were 15–20% lower than the activity of the native lipase (1.66 μmole·h^−1^·mg^−1^), under the same conditions. Such a decrease does not mean inactivation of the enzyme, as distribution of the enzyme inside a porous matrix will certainly increase the weight of the solid preparation and will decrease the specific activity. In order to have an accurate measure of the immobilization process efficiency, the relative total activities were calculated as a ratio of total activity resulted after immobilization and total activity of the enzyme subjected to immobilization. As indicated in Table 1, the results indicate a spectacular increase of catalytic efficiency for the immobilized enzymes, up to 7-fold in case of simple sol-gel entrapment and up to 10-fold in case of the combined method, at the best protein loading values of 11.3 mg/g and 9.5 mg/g, respectively. Comparing the values of Figure 1 and Table 1, results that the sol-gel preparation with the highest activity did not show the highest relative total activity. However, since the differences were not significant, for the subsequent experiments we selected the sample with higher loaded enzyme (entry 3 in Table 1), considering that it was important to use the most active enzyme.

For practical applications, particularly in the pharmaceutical industry, enzyme enantioselectivity is a key issue, since frequently only one enantiomer possesses biological activity, whereas the other is either inactive or toxic. Based on previous results [16], we expected increased enantiomeric excess values of the product obtained in reactions with entrapped lipase. The enantiomeric ratio E is a better expression of enantioselectivity, because it correlates conversion and enantiomer excess values. To be considered efficient for large-scale applications, the enantiomeric ratio E of a biocatalyst must be over 25.

**Table 1:** Influence of enzyme loading on catalytic efficiency of sol-gel entrapped (SG) and entrapped and adsorbed (SGA) CaLB lipase in the acylation of 2-octanol at 40 °C.

Enzyme loading (mg protein/g biocatalyst)		Conversion at 6 h (%)		Activity (μmole·h^−1^·mg^−1^ *)		Relative total activity	
SG	SGA	SG	SGA	SG	SGA	SG	SGA
11.3	9.1	39	29	1.305	0.968	7.23	8.38
15.9	9.5	39	38	1.312	1.238	6.01	10.27
17.4	10.7	44	39	1.410	1.328	6.57	9.10
18.9	11.7	43	37	1.394	1.215	4.85	6.94
23.5	14.0	45	33	1.357	1.081	4.58	5.80

* mg of immobilized enzyme added in the reaction.

The enantioselectivity of sol-gel entrapped preparates was substantially improved compared to non-immobilized lipase (E = 10, under the same reaction conditions). The highest enantiomeric ratio (E = 112) was obtained at 17.4 mg/g enzyme loading. The enantioselectivity of lipase immobilized by the combined method was slightly lower, reaching the highest enantiomeric ratio value (E = 73) at 10.7 mg/g protein loading (Figure 2), but was higher than the value obtained for the native lipase, as well. 

Considering both the activity and enantioselectivity of the obtained biocatalysts, the optimum enzyme loadings were 17.4 mg/g for the sol-gel entrapped lipase, and 10.7 mg/g for the lipase immobilized by the combined entrapment and adsorption method. These values were used in the subsequent studies. 

### 2.2. Ionic Liquids as Templates for Sol-Gel Entrapment of CaLB Lipase

Ionic liquids with 1,3-dialkylimidazolium type cations are mostly used for biocatalytic applications. Their physical properties cover a wide range of values, but the catalytic properties of enzymes are influenced mainly by their polarity, hydrophobicity, and miscibility with the solvent. Polarity and hydrophilicity/lipophilicity can be readily adjusted by a suitable choice of cation/anion and ILs have been referred to as ‘designer solvents’ [27,29]. Ionic liquids have also proved to be effective additives in the entrapment process. However, is difficult to identify their exact role in preparation of sol-gel materials and the influence of their presence during immobilization [17].

In this study, different ionic liquids were tested as immobilization additives for sol-gel entrapment of CaLB lipase using a ternary silane precursors system. The immobilization was performed with PhTMOS, MeTMOS and TMOS silane precursors at 1.6:0.4:1 molar ratio. The activities were measured at 24 h reaction time.

According to Zhou *et al.*, ionic liquids with tetrafluoroborate anions are templates for preparation of mesoporous structures, in which hydrogen bonds between the tetrafluoroborate anion and silanol group of silica gel and π-π interactions between neighboring immidazole groups play an important role in formation of the framework [30]. The ionic liquids can be partially confined in the silica network and may influence the enzyme behavior, leading to a well-ordered mesoporous structure that favors the enzymatic reaction. Apparently, this mechanism can also be applied for the other ILs tested in our experiments, since the activity values were in a close range for all preparations, between 41 and 52%, related to activity of the native enzyme. The only exception was [Emim]BF_4_, leading to a lower activity value that can be explained by the presence of a short alkyl chain in the cationic part (Table 2). Vila-Real *et al.* also observed that ILs affected the structural characteristics of the sol-gel matrix suggesting that they play an important role in enzyme performance [31].

**Table 2:** Influence of IL used as additive on catalytic efficiency of sol-gel entrapped CaLB lipase in enantioselective acylation of racemic 2-octanol, at 24 h reaction time.

Ionic liquid	mg biocatalyst	Conversion (%)	Activity (μmole·h^−1^·mg^−1 ^*)	Relative total activity (%)	ee _P_ (%)
Native lipase(control)	-	20	0.842	-	89
Reference	519.2	48	0.424	2.24	89
(no additive)					
[Emim]BF_4_	617.6	42	0.350	2.16	95
[Hmim]BF_4_	632.6	51	0.432	3.11	95
[Omim]BF_4_	622.8	51	0.429	6.64	95
[Bmim]PF_6_	668.2	51	0.428	3.25	94
[Emim]COOCH_3_	671.7	51	0.432	3.30	93
[Emim]COOCF_3_	720.7	52	0.434	3.56	91
[Bmim]Tf_2_N	702.7	51	0.432	3.45	95

* mg of immobilized enzyme added in the reaction.

**Figure 3:**
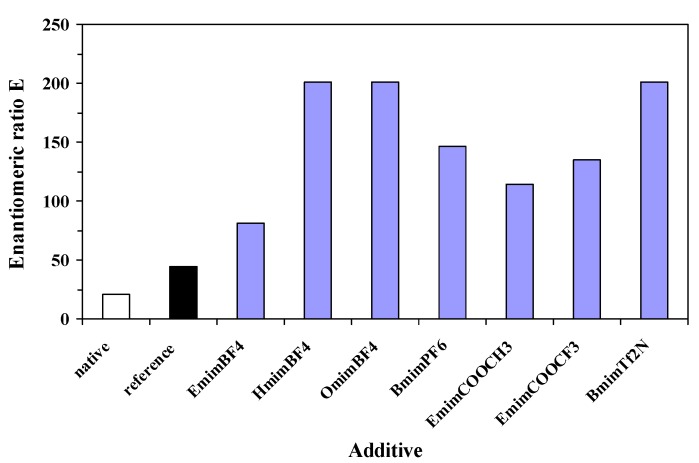
Influence of nature of ionic liquid additive on enantioselectivity of the immobilized lipase, in the enantioselective acylation of 2-octanol, at 24 h reaction time. The reference sol-gel was prepared without any additive.

Like in the results from Table 2, there were no significant differences between the activities of sol-gel preparates obtained with and without ILs, using this immobilization method, but the catalytic efficiency, expressed as relative total activity, increased up to three fold when the best performing IL was used as an additive, due to the higher mass of the immobilized preparation. The explanation is that utilization of an IL leads to increased yield of the sol-gel formation process, resulting in higher xerogel amount. Our immobilized enzymes showed higher enantiomeric excess (between 91% and 95%, Table 2) and enantiomeric ratio E (between 81 and 201, Figure 3) values, compared to the native enzyme (89% and 20, respectively).

Regarding both efficiency criteria, catalytic activity and enantioselectivity, the best performing ionic liquid was [Omim]BF_4_, showing a 6-fold increase of relative total activity and more than 10-fold increase of enantiomeric ratio, compared to non-immobilized lipase.

### 2.3. Stability of Sol-Gel Entrapped Lipase in Organic Solvents

Biocatalysis in organic solvents offers a wide range of advantages compared to aqueous media: better solubility of hydrophobic compounds, possibility to perform reactions that are thermodynamically or kinetically restricted in water, minimization of side reactions, control or modification of enzyme selectivity, simple recovery of the products by using volatile organic solvents and enhanced thermal stability of the enzyme. However, enzymes do not always fulfill the requirements of activity, productivity and, most important, stability in organic solvents. The type of solvent used for a particular type of reaction is essential because it affects both stability and specificity of biocatalysts. It is known that hydrophobic solvents can stabilize and activate the lipases better than the hydrophilic ones, which remove a part of the water that the enzyme needs for its function, leading to a decrease of catalytic activity. Besides influencing the catalyzed reaction, organic solvents can also have denaturing effects on enzymes. Increase of enzyme stability is the main advantage of immobilization, and stability against denaturation caused by physical or chemical factors is an important characteristic of an immobilized enzyme that must be investigated.

The aim was to study the possible denaturation effect of long-term incubation in organic solvents on CaLB lipase immobilized by sol-gel entrapment or sol-gel entrapment combined with adsorption on Celite 545. The immobilization was performed with a ternary silane precursors system of PhTMOS, MeTMOS, and TMOS, at 1.6:0.4:1 molar ratio and [Omim]BF_4_ as immobilization additive. Native and immobilized lipases were incubated for 7 days at 25° C in various organic solvents (*n*-hexane, *i*-octane, cyclohexane, 1,4-dioxane, toluene, methyl-*tert*-butyl ether, tetrahydrofurane, *tert*-butanol, acetone, acetonitrile). Thereafter, the solvents were removed by filtration, the biocatalysts were washed two times with *n*-hexane and their activity was assayed in acylation of 2-octanol with vinyl acetate at 40 °C, in *n*-hexane. 

For most of the tested solvents (excepting DMSO and pyridine), the transesterification activity of the immobilized enzymes was not significantly influenced by nature of the solvent, demonstrating that they did not denature the lipase embedded in the hybrid sol-gel matrix. The ratios between the highest and lowest values were 1.27 for sol-gel entrapped and 1.38 for entrapped and adsorbed lipase. A difference around 30% could be important in large-scale applications, when productivity of the enzyme is a key parameter. Another important conclusion is the availability of numerous organic solvents for synthetic reactions catalyzed by sol-gel entrapped CALB lipase, allowing the chemist to select the best one, in accordance with the specific requirements of the catalyzed reaction. The most important characteristic of a solvent, which can influence the activity, is polarity. This property is not intrinsic and therefore there is no physical quantity that can express it. In general, it is considered that polarity can be best characterized by the dielectric constant. In our case, it was not possible to establish a correlation between the dielectric constant of the solvents and the residual enzymatic activities after 7 days incubation in those solvents. Protic polar solvents (acetone, acetonitrile) showed inactivating effects on the native enzyme, leading to more than 60% decrease of the activity values compared to less polar solvents (*i*-octane, methyl-*tert*-butyl ether) (Table 3). 

**Table 3:** Influence of long-time (7 days) incubation at 25 °C, in different organic solvents, on the enzymatic activity of native, sol-gel entrapped (SG), or sol-gel entrapped and adsorbed (SGA) CaLB lipase, assayed for the acylation of 2-octanol, at 6 h reaction time.

Solvent	Dielectric Constant ^a^	Activity (μmol·h^−1^·mg^−1^)			Enantiomeric Ratio		
		Native	SG	SGA	Native	SG	SGA
*n*-hexane	1.88	1.22	1.44	1.32	7	84	73
*i*-octane	1.94	1.46	1.42	1.24	9	81	68
cyclohexane	2.02	1.13	1.42	1.21	7	84	68
1,4-dioxane	2.25	1.24	1.24	0.92	12	73	38
toluene	2.38	1.11	1.50	1.49	6	125	81
MTBE	2.60	1.61	1.57	1.37	9	146	78
THF	7.58	1.31	1.50	1.44	8	104	81
*tert*-butanol	12.4	0.92	1.58	1.45	6	134	81
pyridine	12.4	0.39	0.36	0.38	2	12	24
acetone	20.7	1.00	1.71	1.50	6	163	92
acetonitrile	37.5	0.89	1.48	1.18	6	98	67
DMSO	46.6	0.04	0.05	0.05	n.d.	n.d.	n.d.

^a^ from reference [32], n.d. - not determined.

For the immobilized preparates high activity values were obtained, without significant differences between less polar and medium-polar solvents. An important observation is that activities of the immobilized preparations are identical or higher compared to the native enzyme. Since the original preparates displayed 15–20% lower activities than the native enzyme, as it was mentioned before, it results that sol-gel immobilization had an important stabilizing effect on CaLB lipase. However, solvents known as having strong denaturant effect on lipases (DMSO and pyridine), maintained this effect also against the immobilized preparations. Despite this, it can be pointed out that immobilization protects the enzyme from inactivation caused by some organic solvents, which could be an important advantage, as allows selection of the reaction medium according to the characteristics of substrates and reaction products. In most cases, sol-gel entrapment led to higher stability in organic solvents than entrapment combined with adsorption, demonstrating that a more compact encapsulation leads to better protection against inactivation caused by organic solvents.

As regarding enantioselectivity, the enzymatic preparates obtained after immobilization showed remarkable enantiomeric ratio (E) values (Table 3). Among the studied immobilization techniques, simple sol-gel entrapment showed the best stability results, in medium polar solvents (acetone, *tert*-butanol, methyl-*tert*-butyl ether), suggesting that these solvents are particularly recommended for kinetic resolutions of secondary alcohols catalyzed by lipase. 

### 2.4. Reuse of the Immobilized Biocatalysts

The reuse of enzymes in multiple reaction cycles is one of the main objectives of immobilization. This is particularly important for highly priced enzymes, because in most cases the cost of enzyme is a key issue for industrial application. Usually, it is considered that an enzyme can be reused until its activity decreases to less than 25% from the initial value.

Numerous research papers focused on improving the stability and reusability of immobilized enzymes, making possible their application at industrial scale. Hara *et al.* proved that lipase Amano PS from *Burkholderia cepacia* immobilized by sol-gel entrapment showed good reusability at room temperature [22]. Similar results were obtained for entrapped *Candida rugosa lipase*, which was reused 5 times at 30 °C [1].

This study was performed with CaLB lipase, immobilized by sol-gel entrapment in a matrix obtained from a ternary precursor mixture of PhMTOS:MeTMOS:TMOS (1.6:0.4:1 molar ratio), using [Omim]BF_4_ as immobilization additive. The same enzyme, immobilized by sol-gel entrapment combined with adsorption on Celite, was also studied, using as reference the native lipase. The reactions were carried out in *n*-hexane, at 60 °C, for 24 h.

**Figure 4:**
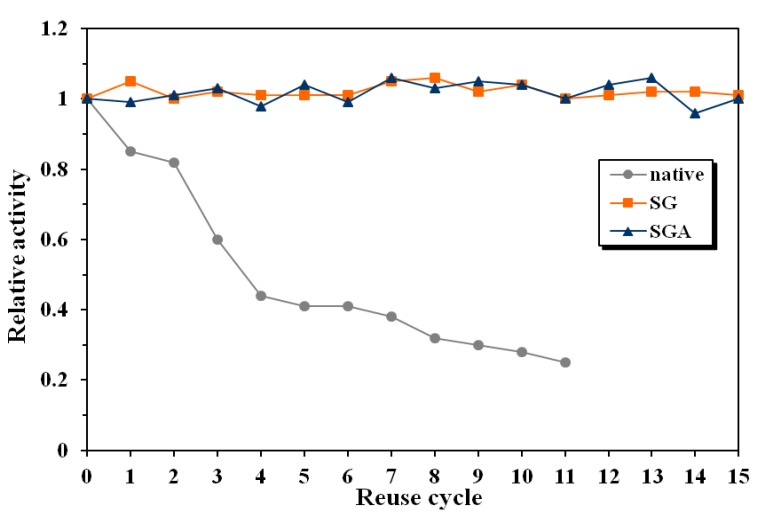
Influence of enzyme reuse on the relative activity of native and immobilized lipase from *Candida antarctica* B, in the enantioselective acylation of 2-octanol at 60 °C in *n*-hexane (native lipase, SG—sol-gel entrapped lipase, SGA—lipase immobilized by sol-gel entrapment and adsorption).

The relative activity of the native lipase decreased with 15% after the first reuse and was only 25% of the initial value after 11 reaction cycles (Figure 4). The conversion value at 24 reaction time decreased from the initial 20% to only 5% after 11 reuses. 

In the same conditions, immobilized lipases maintained their catalytic activity unaltered during 15 reuses. There have not been observed significant differences regarding the operational stability between the employed immobilization methods. The conversion values at 24 h reaction time were between 50% and 52% for both investigated preparates, throughout the whole reuse process. 

The same differences between native and immobilized lipases have been observed for enantioselectivity, as well. The enantiomeric excess of the native enzyme decreased from the initial value of 89% to 63% after 12 reaction cycles. As positive effect of immobilization, the enantiomeric excess values for both tested samples were maintained at the initial 93–94% value during 15 reuse cycles.

Another important conclusion of this study is that catalytic efficiency of the immobilized lipases remained almost unchanged at 60 °C, a high operating temperature for enzymes in organic solvents, while the native enzyme was severely inactivated in the same conditions. This excellent long-term operational stability of sol-gel immobilized enzymes, at higher temperatures than usually employed in enzymatic reactions, is an important argument for industrial use of these biocatalysts.

### 2.5. Morphological Characterization

Physico-chemical characterization of the immobilized enzymes could be a better understanding of their catalytic properties. Scanning electron microscopy (SEM) offers important information concerning the morphology of immobilized biocatalysts. We investigated the enzymatic preparations that previously showed excellent reusability and stability properties.

**Figure 5:**
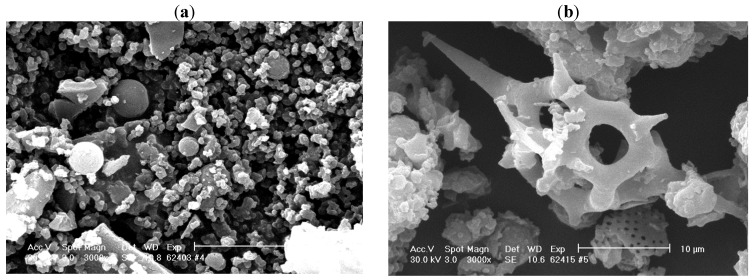
SEM micrographs of (**a**) sol-gel entrapped and (**b**) entrapped and adsorbed CaLB lipase. The sol-gel matrix was obtained with PhTMOS, MeTMOS and TMOS at 1.6:0.4:1 molar ratio and the ionic liquid [Omim]BF_4_ as additive. For the combined immobilization, Celite 545 was employed as adsorbent.

The SEM micrograph obtained for sol-gel entrapped lipase (Figure 5a) showed an amorphous structure with irregular blocks, so we could not identify the pore size or distribution. These results are in agreement with those obtained by Soares *et al.* [33]. When CaLB lipase was immobilized by the combined technique, the SEM image confirms the formation of sol-gel particles on the surface of the solid support, thus partly explaining the high value of the catalytic activity (Figure 5b). According to previously reported results, different gel composites all showed more or less similar morphology, so it is unlikely they play a major role in lipase catalyzed transformations [34].

Fluorescence microscopy offers informations regarding the distribution of the enzyme inside the sol-gel matrix. This analysis can be performed by dying the lipase with a fluorescent compound (fluorescein isothiocyanate-FITC), followed by immobilization as described in the Experimental section. 

**Figure 6:**
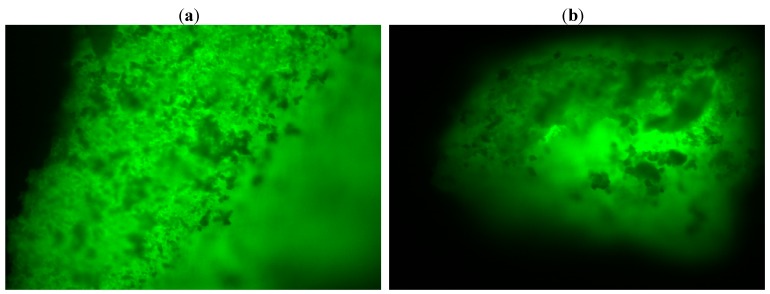
Fluorescent images (10× magnification) of (**a**) sol-gel entrapped and (**b**) double immobilized lipase B from *Candida antarctica*. The sol-gel matrix was obtained with PhTMOS, MeTMOS and TMOS at 1.6:0.4:1 molar ratio and [Omim]BF_4_ ionic liquid as additive. For the combined immobilization, Celite 545 was employed as adsorbent.

A protein free matrix was also obtained and studied. The images are not presented in this work, as the reference material was not fluorescent. The fluorescence microscopy analysis showed an even distribution of the lipase in the sol-gel matrix with no respect to the immobilization technique (sol-gel entrapment or sol-gel entrapment combined with adsorption on Celite 545, Figure 6). Therefore, the high values of total activity cannot be explained only based on a more favorable distribution of the protein in the matrix, the morphology of the biocatalyst should also be taken into consideration. 

## 3. Experimental

### 3.1. General

Lipase from *Candida antarctica* B (CaLB lipase) was produced by C-Lecta (Leipzig, Germany). Silane precursors methyl- (MeTMOS) and phenyl-trimethoxysilane (PhTMOS) were purchased from Merck (Darmstadt, Germany) and tetramethoxysilane (TMOS) from Fluka (Buchs, Switzerland). Other materials used: tris-(hydroxymethyl)-aminoethane (Loba Feinchemie, Fischamend, Austria), 2-propanol (Merck), sodium fluoride (Fluka), Celite 545 (Merck), bovine serum albumin BSA (Sigma-Aldrich, St. Louis, MO, USA), Bradford reagent (Merck), 2-octanol (Merck), vinyl acetate (Merck), *n*-hexane (98%, Merck), acetone (Merck), *iso*-octane 99.5% (Merck), methyl-*tert*-butyl-ether (MTBE) (99.2%, a gift of Rompetrol, Bucharest, Romania), tetrahydrofuran (THF) (Fluka), toluene (Chimopar, Bucharest, Romania), cyclohexane (Fluka), dioxane (Riedel de Haen, Seelze, Germany), acetonitrile (Merck), *tert*-butanol (Merck) were of analytical grade and have been used as purchased. Decane (>99%, Aldrich) was used as internal standard for quantitative gas-chromatographic analysis. Ionic liquids (ILs) 1-ethyl-3-methylimidazolium tetrafluoroborate [Emim]BF_4_, 1-ethyl-3-methyl-imidazolium trifluoroacetate [Emim]COOCF_3_, 1-hexyl-3-methyl-imidazolium tetrafluoroborate [Hmim]BF_4_, 1-butyl-3-methyl-imidazolium hexa-fluorophosphate [Bmim]PF_6_, were purchased from Merck at the highest available purity. 1-Octyl-3-methyl-imidazolium tetrafluoroborate [Omim]BF_4_ was a product of Fluka. Fluorescein isothiocyanate (FITC) was purchased from Sigma-Aldrich.

### 3.2. Immobilization by Sol-gel Entrapment

A microbial lipase suspension (80, 100, 120, 140 or 160 mg/mL) in TRIS/HCl 0.1 M, pH 8.0 buffer was stirred at room temperature for 30 min, centrifuged, and the supernatant used for immobilization. In a 4 mL glass vial, 1 mL of this lipase solution was mixed with 200 µL ionic liquid, followed by addition of 100 µL 1M NaF solution, and 200 µL isopropyl alcohol. This mixture was kept for 30 min under continuous stirring for homogenization, and subsequently a tertiary mixture of silane precursors (total 6 mmol) was added. The mixture was stirred at room temperature until the gelation started. The obtained gel was kept for 24 h at room temperature to complete polymerization. The bulk gel was washed with isopropyl alcohol (7 mL), distilled water (5 mL), isopropyl alcohol again (5 mL) and finally hexane (5 mL), filtered, dried at room temperature for 24 h, and in a vacuum oven at room temperature for another 24 h. Finally, it was crushed in a mortar and kept in refrigerator. The amount of immobilized lipase was determined by measuring the protein content of free enzyme and washing solutions. The protein content was determined using the method developed by Bradford [35]. The immobilization yield was calculated as percentage of encapsulated protein and protein subjected to immobilization. The determined protein content of native lipase was 0.11 mg/mg. Enzyme loadings were expressed as immobilized protein (in mg) related to the weight of the preparate (in g).

### 3.3. Immobilization by Sol-gel Entrapment and Adsorption

The immobilization protocol was identical to that described for the simple sol-gel entrapment, until the start of gelation, when 0.5 g Celite 545 was blended with the gelling mixture. Subsequently, the obtained solid preparate was worked-up as described above. 

### 3.4. Acylation of Secondary Alcohols

Acylations were performed in 4 mL capacity glass vials, charged with a mixture of 2-octanol (0.5 mmole), vinyl acetate (1.5 mmol), reaction medium (organic solvent, 1 mL), decane (internal standard, 15 μL) and free (5 mg) or immobilized lipase (25 mg).

The mixture was incubated using an orbital shaker (MIR-S100, Sanyo, Osaka, Japan) at 300 strokes/min and 40 °C (ILW 115 STD incubator, Pol-Eko-Aparatura, Wodzislaw Slaski, Poland). The conversion and enantiomeric excess of the product were assayed by gas-chromatography, on a Varian 450 instrument (Varian Inc., Middelburg, The Netherlands) equipped with flame ionization detector, using a 30 m × 0.25 mm Elite-Cyclosil B chiral column with 0.25 mm film thickness (Perkin-Elmer, Shelton, CT, USA). The analysis conditions were: oven temperature: 50 °C to 120 °C with 10 °C/min heating rate, injector temperature 240 °C, detector temperature 280 °C, carrier gas (hydrogen) flow 1.2 mL/min. The reactions were usually run for 24 h. Conversions have been calculated based on the internal standard method. 

Transesterification activities were calculated at 6 and 24 h reaction time and expressed as the average 2-acetoxy-alcohol amount (in micromole) synthesized per hour by 1 mg of free or immobilized enzyme. The control reaction without enzyme did not give any product in the same conditions. To characterize the overall efficiency of the immobilization process, relative total activities were calculated as the ratio of total enzymatic activity recovered following immobilization, divided by the total activity of lipase subjected to immobilization. The enantiomeric excess of the resulted ester product (ee_p_) was determined from peak areas of enantiomers, and the enantiomeric ratio (*E*) values were calculated based on conversion and e.e_p_ values using the relation (1) [36]:

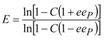
(1)
where *C* represent the conversion at 24 h.

All reactions have been run in duplicate and sampling was also made in duplicate. As the differences between the data for the same assay were less than 2%, average values have been calculated and presented in tables and figures.

### 3.5. Reuse of Immobilized Lipase in a Batch Reaction

The enzyme reuse study was performed at 60 °C. The initial reaction system was set up as described for the acylation study. At the end of every reaction cycle, the product (upper liquid phase of the reaction mixture) was removed with a pipette, the remained solid phase (native or immobilized lipase) was washed two times with 2 mL hexane, centrifuged at 15 °C and 5,000 rot/min, and the supernatant decanted. Subsequently, the same amounts of reagents (2-octanol, vinyl acetate, and hexane) as for the initial reaction were added to the reused enzyme, and the reaction was run under the same conditions. 

### 3.6. Stability in Organic Solvents

The native and immobilized lipases were incubated in organic solvents at 25 °C for 7 days. After this period, the solvent was removed by filtration and the remained solid phase (native or immobilized lipase) was washed two times with 2 mL hexane, centrifuged at 15 °C and 5,000 rot min^−1^, and the supernatant decanted. The activity of biocatalysts was determined in the acylation reaction of 2-octanol with vinyl acetate, as described above. 

### 3.7. Morphological Characterization of the Immobilized Preparates

Scanning electron microscopy (SEM) was performed with an Inspect S +EDAX Genesis XM 2i system (FEI Company, Eindhoven, The Netherlands). The analysis parameters were: pressure 1.5 × 10^−2^ Pa, resolution <10 nm at 3 kV. Fluorescence microscopy was performed with a Leika True Confocal Scanner (Leika TCS SPE, Mannheim, Germany). For this, the lipase was dyed with fluorescein isothiocyanate (FITC) (as described in PIERCE EZ-LabelTM FITC Labeling Kit). The obtained solution (containing the enzyme marked with FITC) was purified on a column and then lyophilized at −55 °C for 34 h. The obtained powder was suspended in TRIS buffer and used for immobilization, using both techniques, sol-gel entrapment and sol-gel entrapment combined with adsorption on Celite 545.

## 4. Conclusions

Considering both activity and enantioselectivity of the obtained biocatalysts, the optimum enzyme loading was 17.4 mg/g for the sol-gel entrapped lipase and 10.7 mg/g for preparations immobilized by the combined method. The best performing ionic liquid as immobilization additive was OmimBF_4_, leading to 6-fold increase of total activity compared to the non-immobilized lipase and high enantiomeric ratio.

Long-term incubation in organic solvents had no damaging effect on the immobilized enzymes, excepting DMSO and pyridine. High residual activity and enantioselectivity values were obtained in solvents with medium polarity, with slightly better results for the preparations immobilized by simple sol-gel entrapment.

The immobilized lipases maintained their catalytic activity unaltered during 15 reuses, without significant differences concerning the operational stability as a result of the immobilization method. An important outcome of this study was the preservation of catalytic activity of the immobilized biocatalysts at 60 °C, in multiple reuse cycles.

Sol-gel entrapped lipase showed an amorphous structure, with irregular blocks. For the sample obtained by entrapment combined with adsorption, SEM image confirmed the formation of sol-gel particles on the surface of the solid support. The fluorescence microscopy analysis showed an even distribution of the lipase in the sol-gel matrix, with no respect to the employed immobilization method.
